# Cutting edge: ESCRT-mediated phagophore closure in mammals

**DOI:** 10.1042/BCJ20250148

**Published:** 2026-04-17

**Authors:** Yoshinori Takahashi, David M. Opozda, Hong-Gang Wang

**Affiliations:** 1 Division of Pediatric Hematology and Oncology, Department of Pediatrics, The Pennsylvania State University College of Medicine, Hershey, PA 17033, U.S.A.; 2 Department of Cell and Biological Systems, The Pennsylvania State University College of Medicine, Hershey, PA 17033, U.S.A.

**Keywords:** autophagosome, autophagy, endosomal sorting, phagophore closure

## Abstract

Autophagy delivers cytoplasmic materials to lysosomes, supporting protein and organelle quality control as well as nutrient recycling to maintain cellular homeostasis. A defining feature of macroautophagy, the major form of autophagy, is the formation of double-membrane autophagosomes that encapsulate cargo either non-selectively or through selective recognition mechanisms. Completion of autophagosome biogenesis requires closure of the phagophore, a step that ensures full cargo sequestration and enables efficient degradation following lysosomal fusion. Recent studies have uncovered a critical role for the endosomal sorting complex required for transport (ESCRT) machinery in mediating phagophore closure, revealing that this event contributes to cellular functions beyond cargo degradation. In the present review, we summarize current advances in defining the molecular mechanisms and physiological significance of phagophore closure in mammals and highlight emerging concepts and future directions for the field.

## Introduction

**Figure 1:**
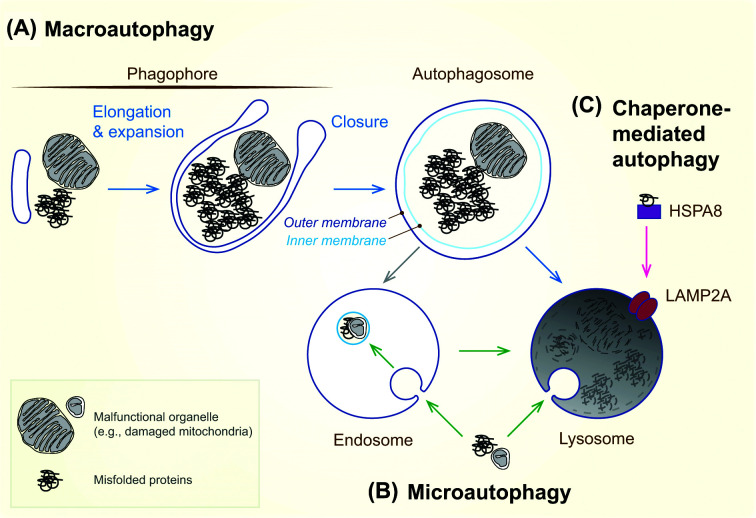
Major autophagy pathways in mammalian cells (**A**) In macroautophagy, cytoplasmic cargoes, including misfolded proteins and malfunctioning organelles such as mitochondria, are enclosed by growing phagophores that eventually close to form autophagosomes. Phagophore closure involves membrane scission, separating the outer and inner autophagosomal membranes. Autophagosomes fuse either directly with lysosomes to form autolysosomes or with endosomes to generate amphisomes, which subsequently fuse with lysosomes. Following endolysosomal fusion, the inner autophagosomal membrane and enclosed cargoes are degraded. (**B**) In microautophagy, cargoes are directly engulfed by lysosomal membranes or sequestered into endosomes that subsequently fuse with lysosomes. (**C**) In chaperone-mediated autophagy, cargo proteins containing KFERQ-like motifs are recognized and unfolded by the chaperone HSPA8 and then translocated across lysosomal membranes via the LAMP2A complex.

Autophagy is a cellular degradation process that delivers cytoplasmic constituents, such as proteins and organelles, to lysosomes for hydrolytic degradation. In mammals, three major types of autophagic pathways have been identified to date: macroautophagy, microautophagy, and chaperone-mediated autophagy [1]. Macroautophagy involves the *de novo* formation of a flat membrane structure, the phagophore, which elongates around cytoplasmic cargo and closes to form a sealed double-membrane autophagosome, which is then delivered to lysosomes (Figure 1A) [2]. In contrast, microautophagy and chaperone-mediated autophagy mediate direct cargo delivery to lysosomes or endosomes (in the case of endosomal microautophagy) via membrane protrusion and invagination [3] or through chaperone–receptor-mediated translocation [4], respectively (Figure 1B,C). Macroautophagy and microautophagy are evolutionarily conserved pathways mediating cargo sequestration in both selective and non-selective manners, whereas chaperone-mediated autophagy, present in most mammalian cell types, selectively targets KFERQ-like motif-containing proteins via the chaperone HSPA8 and the lysosome-associated membrane protein LAMP2A [5].

Among the autophagy pathways, macroautophagy is considered the major route for cargo delivery [6,7] and, for simplicity, will hereafter be referred to as autophagy. The process was first observed in mammalian cells, and the term ‘autophagy’ was coined in 1963 [7,8]. In the 1990s, autophagy was rediscovered in yeast, leading to genetic screens that uncovered its key regulators, the autophagy-related (ATG) genes, many of which were subsequently found to have homologues in other species, including mammals [7,9,10]. Since then, our understanding of the molecular mechanisms and physiological roles of autophagy has greatly advanced. Key machinery includes the ULK1/ATG1 complex, which mediates initiation; the class III phosphatidylinositol 3-kinase complex I, responsible for nucleation; and the LC3/ATG8 conjugation system, together with the ATG2–WIPI complex and ATG9A, which coordinate membrane elongation and expansion. Genetic manipulation of ATG genes in higher eukaryotes, together with the establishment of autophagy markers, such as fluorescent protein-tagged ATG proteins including ATG8/LC3, has demonstrated that dysregulated autophagy-dependent cellular quality control and nutrient recycling contribute to diverse diseases, including cancer, neurodegenerative disorders, metabolic disorders, and immune dysfunction. The details of these machineries and their functional significance have been comprehensively reviewed elsewhere [2,10–16].

Despite advances in understanding the core machinery driving phagophore formation and expansion, the mechanisms governing phagophore closure—the final step in autophagosome biogenesis—remain poorly understood. Although often mischaracterized as a membrane fusion event, phagophore closure is a topologically distinct membrane scission process that transforms the open, single-membrane phagophore into a sealed, double-membrane autophagosome (Figure 1A) [17–19]. In the present review, we highlight recent progress in studying phagophore closure, including the development of robust assays to monitor the process, the identification of the endosomal sorting complex required for transport (ESCRT) membrane scission machinery as a critical regulator, and the potential mechanisms underlying ESCRT targeting and assembly during autophagy. Emerging evidence indicates that the closure event is essential for terminating signaling from molecules recruited to phagophores [20–22]. We further discuss the physiological importance of ESCRT-mediated phagophore closure by reviewing the phenotypes of mice defective in this process.

## Phagophore

The phagophore is a disc-shaped membrane that bends, elongates, and expands into an open ovoid sac as it engulfs cytoplasmic constituents both selectively and non-selectively, eventually sealing the opening to form a double-membrane spherical autophagosome [10,23]. The origin of the phagophore remains incompletely understood, but its rim shows positivity for the β-galactose-binding lectin RCA-120 [24], suggesting an early contribution of post-Golgi membranes during autophagosome biogenesis. At the structural level, the phagophore displays a distinctive feature; the rim is dilated or outwardly recurved, a geometry that may reduce bending energy and stabilize the opening during membrane expansion [23,25–27]. In contrast, the distance between the outer and inner leaflets of its body is markedly smaller than that of other organelles, including mitochondria and the nuclear envelope, and becomes even thinner as the membrane grows, without a corresponding expansion of the intermembrane space [23,26]. The edge of the phagophore frequently contacts the endoplasmic reticulum (ER) or ER-originated, phosphatidylinositol 3-phosphate (PI3P)-enriched membrane compartments (i.e., omegasomes or isolation membrane-associated tubular/vesicular structures) [26,28,29]. These features suggest that phagophore membrane growth is primarily driven by non-vesicular lipid delivery from the ER rather than vesicular fusion. In yeast, the growing phagophore also contacts with the vacuole, a functional equivalent to mammalian lysosomes, potentially allowing autophagosome formation at the same site in rapid succession [26,27]. Membrane contacts between phagophores and endosomes or lysosomes can also be observed in mammalian cells, although these events may be transient, as they are only occasionally detected [30,31]. Besides the ER and endolysosomes, other organelles, including lipid droplets, Golgi, nuclear membrane, and recycling endosomes, have also been reported to form contact sites with, or be located nearby, the phagophore [26,30], suggesting that multiple organelles contribute to autophagosome biogenesis. In selective autophagy, where the phagophore grows around specific cargoes through interactions with cargo receptors (CRs), the cargo itself can serve as a template to scaffold the expanding membrane [12,32]. Additionally, the phagophore can facilitate cargo accumulation by recruiting kinases, such as TBK1, that activate CRs to enhance their cargo binding [22,33]. Thus, the phagophore not only adapts its shape to the cargo but also actively coordinates the recruitment of additional cargoes, ensuring efficient sequestration. These principles may also apply to starvation-induced, ‘non-selective’ autophagy, in which the majority of phagophores are found to form and grow around autophagy receptor p62/SQSTM1-positive condensates [12,34,35]. The closure of the phagophore is the final step in autophagosome biogenesis, in which membrane abscission at the rim separates the outer and inner membranes and seals the structure [12,36]. Notably, this process facilitates, but is not strictly required for, fusion with lysosomes or, in yeast, vacuoles; however, it is critical for efficient cargo delivery and subsequent degradation [36–40].

## Detection of phagophore closure

### HaloTag-ATG8 autophagosome completion assay

**Figure 2:**
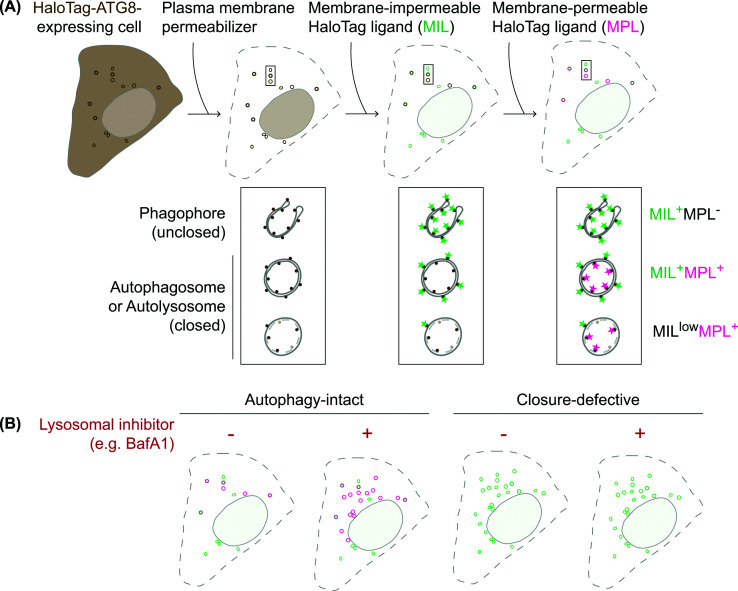
Monitoring phagophore closure using the HaloTag-ATG8 assay (**A**) Cells expressing HaloTag-ATG8 are treated with a plasma membrane permeabilizer to release cytosolic, unconjugated HaloTag-ATG8, leaving only membrane-conjugated HaloTag-ATG8. Cells are then sequentially incubated with MILs and MPLs, each coupled to distinct fluorophores. MILs label HaloTag-ATG8 accessible from the cytosol on unclosed phagophores, whereas MPLs label HaloTag-ATG8 sequestered on the inner membrane of closed autophagosomes and inaccessible to MILs. As autophagosomes mature, ATG8 on the outer autophagosomal membrane is deconjugated, reducing the MIL signal. Fusion with lysosomes diminishes MPL signal due to HaloTag degradation. (**B**) In autophagy-intact cells, lysosomal inhibition leads to accumulation of MPL^+^ structures. In contrast, cells defective in phagophore closure accumulate MIL^+^MPL^-^ structures regardless of lysosomal inhibition.

One of the hallmarks of autophagosome biogenesis is the ubiquitin-like conjugation of ATG8 family proteins (MAP1LC3A, MAP1LC3B, MAP1LC3C, GABARAP, GABARAPL1, and GABARAPL2 in mammalian cells) to phosphatidylethanolamine (PE) on the phagophore—a process known as ATG8ylation—which enables their localization to both the outer and inner leaflets of the membrane. Upon autophagosome closure, ATG8 proteins on the outer leaflet are deconjugated and recycled back to the cytosol, whereas those on the inner leaflet are sequestered within the autophagosomal lumen and delivered to lysosomes for degradation. Leveraging this property in combination with HaloTag labeling technology [41], a fluorescence-based assay was developed to monitor phagophore closure and subsequent autophagosome maturation [36]. HaloTag is a modified haloalkane dehalogenase that covalently binds synthetic HaloTag ligands, which are available in both membrane-permeable and membrane-impermeable forms, labeled with different fluorophores [41]. By permeabilizing the plasma membrane with a cholesterol-complexing agent, such as recombinant perfringolysin (rPFO/XF-MPM) or digitonin, followed by sequential incubation with saturating doses of membrane-impermeable ligand (MIL) and then membrane-permeable ligand (MPL), the assay distinguishes phagophores (MIL^+^MPL^-^), nascent autophagosomes (MIL^+^MPL^-^), and mature autophagosomes/autolysosomes (MIL^low^MPL^+^) (Figure 2A) [36,42,43]. Readouts can be obtained by fluorescence microscopy or fluorescence-activated cell sorting. The assay was originally developed using MAP1LC3B and can be applied to other ATG8 proteins, although their signal intensities vary among paralogs [44], potentially reflecting differences in membrane conjugation and deconjugation. Notably, the principle of the assay can also be applied to assess phagophore closure *in vitro* by incubating acceptor lysates containing HaloTag-LC3-positive phagophores with donor cell lysates in the presence or absence of ATP [45]. One major limitation of the assay is that it may not distinguish ATG8 proteins on phagophores from those on single membranes generated by CASM (conjugation of ATG8s to single membranes), as both appear as MIL^+^MPL^-^ structures. Therefore, to enhance assay specificity, it is highly recommended to perform the assay in the presence or absence of the V-ATPase inhibitor bafilomycin A1 (BafA1), which selectively impairs CASM without affecting autophagosome biogenesis [46]. Since HaloTag sequestered by autophagy can be degraded in lysosomes, leading to diminished MPL signals [36,47], the use of BafA1 to prevent lysosomal acidification also enables monitoring of autophagic flux, as indicated by BafA1-sensitive MPL accumulation. In contrast, cells defective in phagophore closure are expected to display an accumulation of MIL^+^MPL^-^ structures regardless of the presence or absence of BafA1 (Figure 2B). Additional cautionary notes are provided in the recent autophagy guidelines [42].

### Cargo sequestration assays

Prior to the development of the HaloTag-ATG8 assay, cargo sequestration assays and electron microscopy were the two major approaches used to assess phagophore closure. In cargo sequestration assays, crude cellular membranes separated from the cytosol are subjected to proteinase K treatment, and the protease sensitivity of membrane-conjugated ATG8 proteins or autophagic substrates (e.g., p62/SQSTM1) is measured [48,49]. Alternatively, the crude membrane fraction can be subjected to a freeze-thaw cycle to release cytosolic enzymes (e.g., lactate dehydrogenase) nonselectively sequestered within autophagosomes, followed by measurement of their enzymatic activity [50,51]. If closure is defective, ATG8 and cargo molecules should be sensitive to protease digestion in the former assay, whereas enzyme activity should be lost in the latter. Major limitations of cargo sequestration assays include their technical complexity and potential confounding from autophagy-independent ATG8ylation or cargo sequestration (e.g., endolysosomal microautophagy). However, they have the advantage of being applicable to tissue samples without the need for reporter expression. More recently, using the LOVTRAP system for photoinduced protein dissociation, an optogenetic closure assay was developed to monitor autophagic sequestration of damaged mitochondria [52]. This assay requires transgenes but enables monitoring of phagophore closure in live cells and could potentially be extended for real-time monitoring of other autophagic cargoes, such as p62, during starvation-induced autophagy.

### Electron microscopy

Electron microscopy is considerably more labor-intensive compared with the assays described above. Additionally, the phagophore opening at the closure stage is expected to be very small [52], which could make the detection of closure defects challenging, even when reconstructing 3D volumes using electron tomography. Nonetheless, accumulation of phagophores has been observed in electron micrographs of cells defective in ATG8ylation [53,54] or ESCRT-mediated membrane scission [36,43]. Notably, osmium fixation in the absence of potassium ferrocyanide artificially enlarges the intermembrane space of phagophores and autophagosomes [55], allowing immature autophagic structures, including phagophores, to be readily detected [36,43]. Given that both the HaloTag-ATG8 assay and cargo sequestration assays can potentially detect non-autophagic activities, as described above, electron microscopy remains a critical approach for validating phagophore closure defects.

## Mechanism of mammalian phagophore closure

### The ATG8 and its conjugation machinery

ATG8ylation is a ubiquitin-like conjugation process initiated by the E1-like ATG7, transferred by the E2-like ATG3, and completed by the E3-like ATG12–ATG5/ATG16 complex [56,57]. The cysteine protease ATG4 cleaves the C-terminus of newly synthesized ATG8 to expose a glycine for conjugation and mediates ATG8–PE deconjugation for recycling. Electron micrographs of cells with disrupted ATG8ylation, either by replacing endogenous ATG5 with a conjugation-defective mutant, overexpressing an inactive ATG4B mutant, or depleting ATG3, have revealed the accumulation of small, crescent-shaped membranes [53,54,58]. Moreover, time-lapse analysis of soluble N-ethylmaleimide-sensitive factor attachment protein receptor syntaxin 17-labeled autophagosomal membranes shows the impairment of oblate-to-spherical morphological change by ATG3 loss [37]. These studies collectively indicate a critical role for ATG8ylation in phagophore closure.

The mechanisms by which ATG8ylation regulates phagophore closure in mammalian cells remain largely unknown. *In vitro*, ATG8 proteins exhibit liposome tethering and fusion activities [59–61], suggesting that intrinsic membrane-deformation properties may contribute to closure. However, these activities alone are insufficient, as ESCRT inhibition impairs phagophore closure without impairing ATG8ylation [36,43–45,52,62]. An alternative possibility is that ATG8 proteins help recruit or assemble the closure machinery. Each ATG8 protein contains a docking site for LC3-interacting region (LIR) or Atg8-family interacting motif sequences, enabling the recruitment of diverse effectors such as biogenesis regulators, CRs, and signaling molecules to phagophores [56,63]. In plants, such interactions have been shown to facilitate the recruitment of membrane-remodeling machinery during autophagy [64,65]. Moreover, as discussed below, several ATG8-binding proteins have emerged as potential regulators of mammalian phagophore closure [43,45]. Together, these observations support a model in which ATG8 proteins coordinate the recruitment and/or assembly of the closure machinery, thereby facilitating autophagosome completion.

### The ESCRT machinery

**Figure 3:**
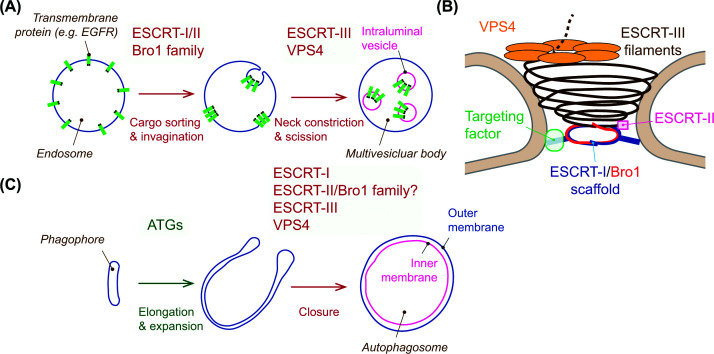
ESCRT-mediated membrane scission (**A**) ESCRT silences endocytosed signaling receptors such as epidermal growth factor receptor (EGFR) by sorting them into intraluminal vesicles (ILVs), forming multivesicular bodies. ESCRT-0 (not shown) recognizes ubiquitinated receptors and PI3P, targeting the downstream ESCRT machinery, which then coordinates cargo sorting, membrane invagination, constriction, and scission to generate ILVs. (**B**) At the membrane neck, ESCRT-I and Bro1 family proteins assemble into helices that scaffold ESCRT-III filament formation. ESCRT-I engages ESCRT-III via ESCRT-II, while Bro1 family proteins bind directly to ESCRT-III. VPS4 uses its ATPase activity to remodel ESCRT-III filaments, driving neck constriction, membrane scission, and filament disassembly. (**C**) During autophagy, the phagophore elongates and expands to enwrap cytoplasmic cargo and is ultimately sealed to form an autophagosome. ATG proteins coordinate phagophore growth, while ESCRT mediates membrane scission at the phagophore opening, separating the outer and inner autophagosomal membranes to complete closure. The factors that target ESCRT to the phagophore closure remain to be clarified.

The ESCRT machinery is an assembly of protein subcomplexes that mediates membrane remodeling by driving ‘reverse-topology’ scission away from the cytosol. Four core components comprise the machinery: ESCRT-I, ESCRT-II, ESCRT-III, and the AAA ATPase VPS4, which are sequentially recruited to target sites. Canonically, ESCRT-I initiates machinery recruitment by recognizing site-specific factors and forms a helical assembly to scaffold ESCRT-III filament formation at the membrane neck [66,67]. By forming a supercomplex with ESCRT-II, ESCRT-I can also induce membrane invagination. ESCRT-II bridges ESCRT-I and ESCRT-III. Unlike ESCRT-I and ESCRT-II, which form stable protein complexes, ESCRT-III is transiently assembled into filaments on target membranes [68–70]. VPS4 mediates the remodeling and disassembly of ESCRT-III filaments, enabling membrane scission and recycling of the components (Figure 3A,B). In certain membrane remodeling processes, Bro1 family proteins, including ALIX, can functionally substitute for ESCRT-I and/or ESCRT-II [71–73]. Since the discovery of its role in sorting ubiquitinated endosomal cargoes into multivesicular bodies [74], the functions of the ESCRT machinery have expanded to a wide range of cellular processes, including cytokinesis, nuclear envelope sealing and quality control, and plasma membrane and lysosomal membrane repair [75].

As a membrane remodeling event, phagophore closure is topologically equivalent to ESCRT-mediated endosomal sorting (Figure 3C) [17–19], and earlier studies demonstrated the accumulation of autophagic structures upon ESCRT inhibition [18,76–79]. Due to technical limitations, it had long remained unclear whether these structures were unclosed or closed [80]; however, the recent advances in phagophore closure-monitoring assays described above now allow for the robust detection of phagophore closure defects and have demonstrated the critical role of ESCRT in phagophore closure [36,52]. Through HaloTag-LC3B-assay-based focused siRNA and genome-wide CRISPR screens, the ESCRT-III subunit charged multivesicular body protein (CHMP) 2A and the ESCRT-I subunit VPS37A were first identified as critical factors for phagophore closure [36,43]. To date, additional ESCRT components, including TSG101 and VPS28 (ESCRT-I) [43,66], VPS36 (ESCRT-II) [81], CHMP4B (ESCRT-III) [52,82], CHMP3 (ESCRT-III) [36,83], CHMP2B (ESCRT-III) [81,83,84], CHMP1A/B (ESCRT-III) [82], CHMP7 (ESCRT-III) [36], and VPS4A/B [36,52], have also been shown to be involved in the closure process. Among these ESCRT factors, VPS37A is distinguished by its selective requirement for autophagy over other membrane remodeling processes mediated by ESCRT, including endosomal receptor sorting [43,85]. Notably, while ESCRT-I containing VPS37A is required for phagophore closure, it is dispensable for cup-shaped phagophore formation (Figure 3C) [43,66,85]. ESCRT appears to remain important after phagophore closure by maintaining the membrane integrity of autophagosomes [45].

### VPS37A, a key mediator of ESCRT targeting during autophagy

**Figure 4:**
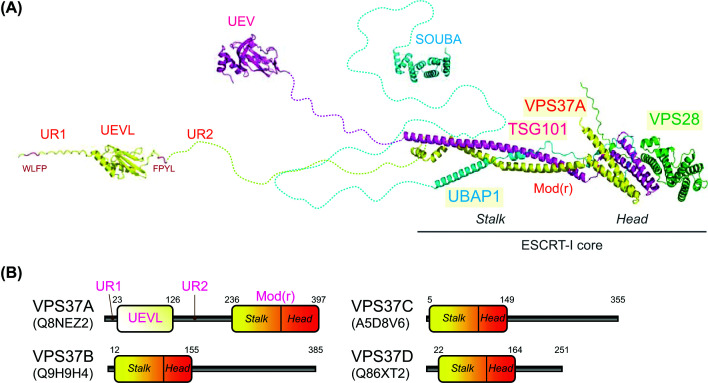
Structural organization of the human VPS37A-containing ESCRT-I complex and VPS37 paralogues (**A**) AlphaFold3 [186]-predicted structure of the human VPS37A-containing heterotetrameric ESCRT-I complex, composed of VPS37A (yellow), TSG101 (magenta), VPS28 (green), and UBAP1 (cyan). ESCRT-I core (stalk and head), TSG101 ubiquitin E2 variant domain (UEV), VPS37A UEV-like domain (UEVL), and UBAP1 solenoid of overlapping ubiquitin-associated domains (SOUBA) were modeled separately and manually connected via the corresponding linkers. Hydrophobic WLFP and FPYL motifs within the unstructured regions (UR1/2) of the VPS37A N-terminus that sense high membrane curvature are highlighted in red. Mod(r) (VPS37A), modifier of rudimentary. (**B**) Domain organization of human VPS37 paralogues (VPS37A–D).

Mammalian ESCRT-I is a heterotetrameric complex composed of TSG101, VPS28, one of the VPS37 paralogues (VPS37A–D), and a UBAP1–MVB12A-associated (UMA) domain protein (MVB12A/B, UBAP1, or UMAD1) (Figure 4A) [36,66]. The pairing of VPS37 and UMA domain subunits in the complex is highly selective: the VPS37A-containing complex incorporates UBAP1, whereas the complexes containing VPS37B or VPS37C incorporate MVB12A/B or UMAD1 [86,87]. The VPS37D-containing complex is the least studied, although a weak interaction between VPS37D and UMAD1 has been observed [86]. All VPS37 proteins contain a modifier of rudimentary (Mod(r)) domain that comprises the stalk followed by the headpiece, the core-forming elements of the ESCRT-I complex, and is responsible for the selective incorporation of a UMA protein (Figure 4B) [66,87–89]. The ubiquitin E2 variant (UEV) domain of TSG101 and the adjacent proline-rich linker, connected at the terminal end of the stalk, mediate the recognition of ubiquitinated cargoes, site-specific adaptor proteins, and ALIX for subsequent ESCRT-III assembly in various ESCRT-mediated membrane remodeling processes, although their role in autophagosome closure remains unknown. The headpiece is capped by VPS28 and mediates ESCRT-I oligomerization and ESCRT-II recruitment for ESCRT-III assembly. UMA domain proteins recognize membranes, ubiquitinated cargoes, and other proteins and are believed to provide selectivity in target recognition within ESCRT-I complexes [90]. Notably, while UMA protein incorporation is required for endosomal EGFR sorting [87] and full HIV-1 infectivity [91], it appears to be dispensable for phagophore closure [22].

The VPS37 paralogues can function both redundantly and non-redundantly within ESCRT pathways. In the endosomal sorting pathway, VPS37A and VPS37B act redundantly to regulate the down-regulation of EGFR and its associated signaling [85], although the VPS37A-containing complex appears to play a dominant role, potentially due to the presence of UBAP1, which harbors SOUBA mediating ubiquitin recognition [87,92]. In contrast, VPS37A is required for phagophore closure, whereas VPS37B and likely other paralogues are not [43,85]. This selective requirement appears to depend on its uniquely extended N-terminal region [43], which comprises a ubiquitin E2 variant-like (UEVL) domain flanked by two unstructured regions (UR1/2) (Figure 4A,B) [93]. Each of the unstructured regions contains a hydrophobic membrane-binding motif that senses highly curved membranes and is important for ESCRT-I targeting during phagophore closure [93]. The N-terminal side of the unstructured region (UR1) also contains a putative LIR and interacts with a subset of ATG8 proteins, specifically GABARAP and LC3A [45]. The loss of ATG8s impairs VPS37A recruitment to autophagosome formation sites [44], further supporting their roles in ESCRT targeting during autophagy. Together, these findings suggest a model in which the highly curved phagophore rim and ATG8 proteins cooperate to mediate ESCRT recruitment. Notably, however, neither the deletion nor mutations of VPS37A UR1 are sufficient to impair phagophore closure and subsequent autophagic flux [22,44,93], indicating that additional mechanisms must underlie ESCRT recruitment. A key element for understanding such mechanisms could be the VPS37A UEVL domain, whose deletion impairs phagophore closure without overt disruption of ESCRT-I complex formation or EGFR sorting [22]. Despite the structural similarity between the VPS37A UEVL domain and the TSG101 UEV domain, they lack amino acid sequence homology and are not interchangeable in autophagy [93,94]. Moreover, unlike the TSG101 UEV domain, the VPS37A UEVL domain lacks the ability to bind ubiquitin [93,94]. Thus, the VPS37A UEVL domain may function to specifically recognize autophagy-related adaptor molecules, enabling ESCRT-I recruitment to phagophores. Interestingly, the CRISPR screen for phagophore closure regulators described above also identified ectopic P-granule autophagy protein 5 (EPG5), in addition to VPS37A and CHMP2A [43]. EPG5 is a large, coiled-coil domain-containing, shepherd’s-staff–shaped protein that has been shown to function as a tethering factor by interacting with ATG8 proteins on autophagosomal membranes and RAB7 on endosomal membranes [95,96]. Proximity proteomics of LC3B-conjugated phagophores accumulated in starved VPS37A-deficient cells has revealed enrichment of EPG5 and RAB7 along with autophagosome biogenesis regulators and autophagic CRs [22], suggesting additional roles of EPG5, such as acting as an autophagic adaptor for ESCRT-I recruitment beyond its tethering function. Future studies are warranted to further define the mechanisms by which VPS37A directs ESCRT recruitment during autophagy.

### ESCRT-III assembly and VPS4-dependent membrane abscission during autophagy

Coarse-grained simulations of ESCRT assembly at HIV-1 budding sites suggest that ESCRT-I is gradually recruited to the Gag lattice and stabilized by forming helical assemblies when the membrane neck narrows [97], serving as a geometry-dependent checkpoint that provides a template for downstream ESCRT-III filament formation [66]. In autophagy, mutation of VPS28 helical interface residues does not impair initial ESCRT-I recruitment to the phagophore but blocks its stabilization and phagophore closure [66], indicating a similar scaffolding role for ESCRT assembly. ESCRT-II, composed of one copy each of VPS36 and VPS22 and two copies of VPS25, can bridge the ESCRT-I and ESCRT-III complexes through interactions with VPS28 and the myristoylated ESCRT-III protein CHMP6, which in turn promotes CHMP4 recruitment [98]. During autophagy, VPS36 is targeted to the phagophore opening via interaction with PI3P and its depletion causes VPS37A to distribute more diffusely across phagophores compared with wild-type cells [81]. Thus, ESCRT-II may also promote the concentration of ESCRT-I at the phagophore closure site, in addition to bridging ESCRT-I and ESCRT-III. However, loss of VPS25, which also destabilizes VPS22, is not sufficient to block phagophore closure or autophagic flux [43]. The lack of ESCRT-II or Bro1 family genes among the hits from the CRISPR screen for phagophore closure regulators [43], despite their dispensability for cell survival, suggests that additional factors cooperate to mediate ESCRT-III filament assembly during autophagy. Interestingly, in addition to ESCRT-I, ALIX, which can directly interact with CHMP4 proteins [99], has also been shown to form helical assemblies. In cells, CHMP4B filaments were found intertwined with co-assembled ESCRT-I and ALIX helical filaments [72], suggesting a mechanism for ESCRT-III assembly that can bypass the requirement for ESCRT-II (Figure 3B). CHMP7, which contains a VPS25-like domain and an ESCRT-III-like domain [97], may likewise support ESCRT-III assembly independently of ESCRT-II during autophagy, as transient CHMP7 depletion leads to a mild accumulation of phagophores [36].

Mammalian ESCRT-III is composed of eight distinct members: CHMP1–7 and IST1. Each ESCRT-III protein can transition between a closed, inactive, soluble monomer and an open, active, membrane-bound oligomer, the latter of which assembles into homo- or heterofilaments. The active conformational change is initiated by site-specific membrane-resident binding partners, which serve as nucleation seeds, and subsequently recruits additional ESCRT-III subunits through inter-subunit interactions to drive filament assembly at target membranes. CHMP4, CHMP3, and CHMP2 appear to serve as core filament components, with the remaining ESCRT-III proteins assisting in filament assembly and functional regulation [100,101]. Several models have been proposed for ESCRT-III-mediated membrane abscission, but the current consensus is that ESCRT-III-mediated membrane abscission occurs through a dynamic, ATP-dependent subunit-exchange mechanism driven by VPS4 [68,100–102]. In the present model, filament assembly is initiated by CHMP4 and rigidified by CHMP2–CHMP3 heterofilaments. VPS4, with the support of another ESCRT-III-interacting protein, VTA1, mediates the incorporation and removal of ESCRT-III subunits, driving filament remodeling, geometric transitions, and membrane constriction that ultimately leads to membrane fission and filament disassembly for component recycling. Inhibition of VPS4 by the overexpression of an ATPase-deficient dominant-negative mutant of VPS4A results in the accumulation of CHMP2A on LC3-positive phagophores and impairs phagophore closure [43], aligning with the role of VPS4 ATPase-driven ESCRT-III filament remodeling in autophagy. Interestingly, a recent study demonstrated that CHMP2A is recruited to the phagophore by interacting with ATG9A through the adaptor protein IQGAP1 [103]. Thus, besides interacting with CHMP4B [104], CHMP2A activation can be controlled by autophagy-specific adaptor-mediated interactions.

### Other factors involved in phagophore closure

**Figure 5:**
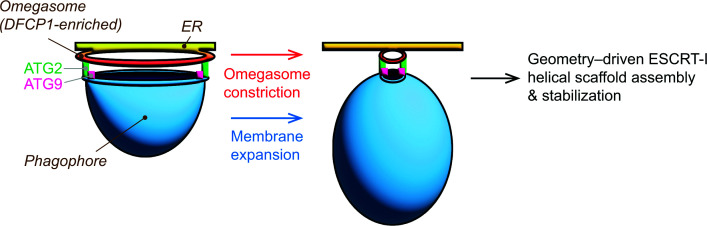
Omegasome constriction and membrane expansion as potential regulators of ESCRT assembly The growing edge of the phagophore associates with the PI3P-enriched ER subdomain known as the omegasome, from which ATG2 transfers lipids to the phagophore. ATG9 scrambles lipids across the phagophore bilayer to promote membrane expansion, while the ATPase DFCP1, a PI3P effector enriched on the omegasome, drives omegasome constriction in an ATPase-dependent manner. Together, membrane expansion and omegasome constriction narrow the phagophore opening, which in turn triggers stable assembly of the ESCRT-I helical scaffold to template ESCRT-III filament formation for phagophore closure.

In addition to ATG8s and ESCRTs, ATG2 and its interacting protein TRAPPC11 have been reported to be involved in phagophore closure [49,105,106]. Although the precise mechanism by which ATG2 regulates phagophore closure remains unclear, ATG2 is a lipid transfer protein that functions together with ATG9 to drive phagophore expansion [80,107]. Phagophores accumulated in ATG2A/B-deficient cells appear to be much smaller than those observed in ESCRT-deficient cells [43,108–110]. Thus, inhibition of phagophore growth may indirectly impair ESCRT-dependent phagophore closure by preventing the phagophore from reaching a geometry favorable for ESCRT-I oligomerization. In line with this, DFCP1, a PI(3)P effector protein located at the omegasome [111], functions as a large ATPase to regulate the constriction of phagophore opening [112,113]. It is conceivable that DFCP1 acts upstream of ESCRT-mediated closure, and future studies are warranted to test this possibility (Figure 5).

Cytosolic calcium ions (Ca^2+^) are another factor that can potentially impact phagophore closure. Local increases in Ca^2+^ concentration induced by the calcium ionophore A23187 and the SERCA inhibitor thapsigargin have been shown to impair autophagic cargo sequestration [114] and subsequent lysosomal fusion [115], while phagophore formation and expansion appear to be unaffected [115–117]. Notably, in certain ESCRT-mediated membrane remodeling processes, including plasma membrane repair, lysosome repair, and microautophagy, Ca^2+^ acts as a signal to trigger ESCRT-III assembly via the Ca^2+^-binding sensor ALG2, which recruits ALIX and/or TSG101 [118–121]. Local accumulation of the ESCRT machinery has been observed in cells treated with the calcium ionophore ionomycin and thapsigargin [118,121,122]. Such accumulation at non-autophagic sites may limit the availability of ESCRT components at the phagophore, thereby impairing membrane closure.

Phase-separated biomolecular condensates may also play an important role in phagophore closure. In plants, the Ca^2+^-dependent lipid-binding protein 1, recruited to the phagophore via interactions with PI3P and ATG8, has been shown to coassemble with ALIX into biomolecular condensates, which may facilitate ESCRT-III filament formation for phagophore closure [64]. Interestingly, during multivesicular body biogenesis, the plant-specific ESCRT component FREE1 can also undergo phase separation, which not only promotes ESCRT recruitment but also drives spontaneous membrane scission independently of the core ESCRT machinery [123]. Such membrane-remodeling ability may contribute to residual autophagic activity in ESCRT-depleted cells [36,43,52,66].

In addition to mammals [22,36,52], the ESCRT machinery has been shown to mediate phagophore closure in other species, including yeast (*Saccharomyces cerevisiae*) [124] and plants (*Arabidopsis thaliana*) [65]. In these organisms, VPS37 homologues appear to lack the unique N-terminal region found in mammalian VPS37A, suggesting that the molecular mechanisms responsible for ESCRT recruitment during autophagy may differ between species. Indeed, in yeast, ESCRT-III is directly targeted to the phagophore through interaction with Atg17 [124], a functional counterpart of mammalian FIP200 that lacks sequence homology [125,126]. In plants, ESCRT-III targeting to the phagophore requires FREE1, whose homologue is absent from mammalian cells [65]. Accumulation of LC3-II (PE-conjugated form), p62, and ubiquitinated proteins were detected in various tissues of the VPS37A mutant mice [22] described below, suggesting a general requirement for VPS37A in mammalian autophagy. Nonetheless, it remains possible that VPS37A-independent alternative pathways also exist in certain types of mammalian cells to mediate ESCRT-dependent phagophore closure.

## Roles of phagophore closure as a signaling checkpoint

**Figure 6:**
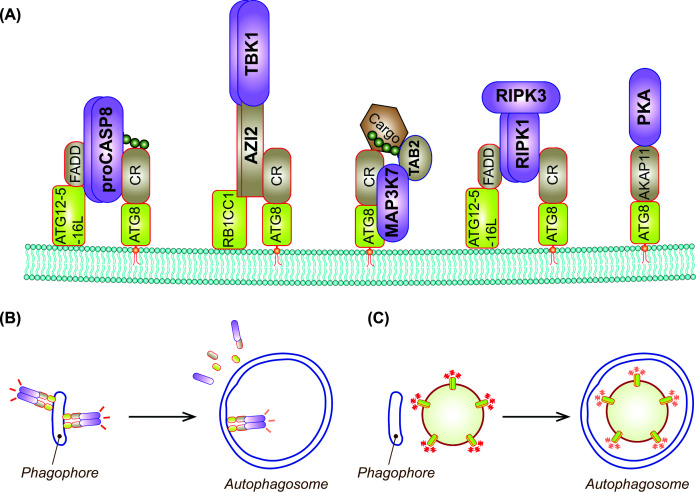
Phagophore closure regulates diverse signaling pathways (**A**) Signaling molecules can be recruited to autophagosome formation sites or phagophores through interactions with CRs, ATG8 family proteins, and other autophagy regulators. (**B**) Upon autophagosome completion, signaling molecules on the concave side of the phagophore become encapsulated, whereas those on the convex side are released through dissociation of ATG proteins; both outcomes silence silencing. (**C**) Phagophore closure can also silence additional signaling compartments, including hypersignaling endosomes.

During autophagy, various signaling molecules can be targeted to autophagosome formation sites through interactions with CRs, ATG8 family proteins, and other autophagy regulators (Figure 6A). Such recruitment has been shown to promote the activation of certain molecules, including those that undergo proximity-induced trans-activation, such as the apoptosis initiator CASP8 [127–131], the innate immune and autophagic signaling kinase TBK1 [132–137], the stress- and inflammatory signaling kinase TAK1/MAP3K7 [138,139], and the necroptosis kinases RIPK1/3 [140–143]. Additionally, the ACAP11-PKA holocomplex has been demonstrated to be scaffolded to ATG8ylated membranes to modulate downstream signaling [144]. The activation of these molecules and their associated signaling pathways is likely stabilized when phagophore closure is inhibited, which not only prevents their encapsulation and subsequent degradation, but also blocks the disassembly of signaling complexes by inhibiting the dissociation of autophagy regulators, including ATG8s (Figure 6B). Supporting this theory, blocking phagophore closure by depleting CHMP2A or VPS37A stabilizes CASP8 assembly and primes cells for apoptosis [20,21]. Enhanced accumulation of active TBK1 and its adaptor AZI2 on LC3- and p62-double-positive structures is also observed in starved, VPS37A-deficient, or VPS37A UEVL mutant cells [22]. Apart from its role in innate immune signaling, TBK1 is well-known for initiating autophagosome formation by interacting with and phosphorylating autophagy receptors, thereby enhancing cargo condensation and recruitment of the core autophagy machinery [145,146]. The TBK1 complex also can interact with RB1CC1/FIP200, a component of the autophagy initiation ULK1 complex, and ATG8 family proteins [147]. Interestingly, although CR condensation can occur independent of ATG8ylation [34], disruption of ATG8ylation mitigates TBK1-dependent p62 phosphorylation and condensate formation in VPS37A-deficient cells, suggesting a role for ATG8ylation in efficient cargo accumulation [22]. By coordinating autophagosome formation and cargo condensation, TBK1 at the autophagosome formation sites may also influence the activation of other signaling molecules described above.

Defects in ESCRT-dependent endosomal sorting can cause aberrant receptor signaling, contributing to cancer progression [75]. A recent study demonstrated that autophagic sequestration of such hyperactive signaling endosomes is important for terminating their signaling (Figure 6C). Loss of VPS37A impairs this process, resulting in sustained EGFR signaling and enhanced cell migration [85]. Thus, beyond its role in autophagic degradation, phagophore closure may act as a signaling checkpoint that responds to defects in the endosomal pathway.

## Physiological impact of disrupting ESCRT-dependent autophagy

Systemic impairment of autophagy through disruption of ATG8ylation results in neonatal death in mice due to a lack of self-derived nutrient supply and impaired milk intake [14]. This neonatal lethal phenotype can be rescued by restoring ATG8ylation specifically in neurons [148], underscoring the essential role of neuronal autophagy in early postnatal survival. The critical role of autophagy in neurons is further demonstrated in adults, where disruption of ATG8ylation leads to neurodegeneration [149–151]. Notably, disruption of the ESCRT machinery also causes neurodegeneration, and mutations in genes encoding its components, such as CHMP2B and CHMP3, have been reported in human neurodegenerative diseases, including frontotemporal dementia and amyotrophic lateral sclerosis. In these neuronal cells, accumulation of autophagic structures, in addition to aberrant endosomes, has been observed [78,79,152–154], indicating the critical role of ESCRT in neuronal autophagy.

To dissect the role of ESCRT in phagophore closure and more precisely assess the physiological impact of impaired phagophore closure, mice carrying a VPS37A UEVL deletion were generated. In contrast with endosomal sorting–defective mice lacking HGS, TSG101, or the UBAP1 SOUBA domain, which are embryonic lethal [155–158], VPS37A mutant mice were born at the expected Mendelian ratio and, aside from a slightly reduced body size, did not display overt developmental abnormalities. Nonetheless, approximately half of the mice died shortly after birth, and all surviving mice developed neurological abnormalities, ultimately reaching the experimental endpoint within three months of age [22]. Together, these studies highlight the importance of ESCRT-mediated phagophore closure in neuronal maintenance. In addition to neurological defects, VPS37A mutant mice also display tissue abnormalities observed in autophagy-deficient mice [14,22,148,149]. However, the overall phenotypes appear milder than those seen in mice lacking core ATG genes [14]. The basis of these milder phenotypes remains unclear. Residual autophagic activity detected upon inhibiting ESCRT-dependent phagophore closure [36,43,52,66] may contribute to the relatively mild phenotypes. At the cellular level, however, the rate of bulk, non-selective autophagic degradation is comparable between VPS37A UEVL mutant and ATG7-deficient cells [22]. Moreover, mice deficient in EPG5 also develop neurological abnormalities, yet their overall phenotypes appear to be milder than those of ATG-deficient mice [159,160].

One plausible factor contributing to the milder phenotypes observed in VPS37A UEVL mutant mice is the accumulation of ATG8ylated membranes resulting from impaired phagophore closure. These ATG8ylated membranes appear to enhance TBK1-dependent cargo clustering and enwrapping, potentially mitigating cytotoxicity associated with defects in autophagic clearance [161]. Supporting this idea, in flies, neuronal loss caused by ESCRT-III dysfunction can be suppressed by overexpression of Ik2, the fly homologue of TBK1, whereas inhibition of Ik2 exacerbates this degeneration [162]. Notably, however, in cultured rat cortical neurons, disruption of ATG8ylation does not enhance, but rather suppresses cell death caused by ESCRT-III dysfunction [163]. Moreover, in osteosarcoma cells, inhibition of ATG8ylation mitigates CHMP2A depletion-induced, CASP8-dependent cell death [21]. Thus, through recruitment of TBK1 and CASP8, ATG8ylated phagophores may act as context-dependent signaling platforms, either promoting cell survival or triggering cell death. The factors that dictate these context-dependent outcomes are not yet fully understood, but autophagy-independent signals, including ER stress triggered by ESCRT-III dysfunction, appear to direct ATG8ylated phagophores toward pro-death outcomes [20].

Another potential contributing factor is the autophagy-independent functions of VPS37A, which may be compromised in VPS37A UEVL mutant mice. Recent studies have revealed roles for the VPS37A-containing ESCRT-I complex in microautophagy of stimulator of interferon genes vesicles and tau aggregates [164–166]. Interestingly, VPS37A, together with UBAP1 and other ESCRT components, has been implicated as a macroautophagy-independent regulator of NCOA4 turnover [167]. NCOA4 is a ferritin-interacting protein that drives ferritin condensation, facilitating its degradation for iron recycling through macroautophagy and microautophagy [168]. Iron released through ferritin degradation has been shown to promote ferroptosis [169,170], an iron-dependent form of regulated cell death [171]. Defects in ferritin degradation due to impairment of both autophagy pathways may alter cellular iron homeostasis and influence susceptibility to ferroptotic cell death. Whether the VPS37A UEVL domain is involved in regulating microautophagy remains an important question for future investigation.

## Future perspectives

Recent advances in distinguishing unclosed phagophores from closed autophagosomal structures have enabled robust analysis of phagophore closure, illuminated the once-opaque closure mechanism, and established the ESCRT machinery as its core regulator [36,43,45,52,62,66,124]. Among mammalian ESCRT components, VPS37A has emerged as a critical factor selectively required for phagophore closure over endosomal receptor sorting [43,85]. This unique requirement maps to its N-terminus, which comprises a UEVL domain flanked by curvature-sensing, unstructured regions and is responsible for ESCRT-I targeting during autophagy [22,43,45,93]. On the phagophore, ESCRT-I forms a helical assembly that may serve as a template for ESCRT-III filament formation [66]. Emerging evidence also indicates that non-ESCRT factors contribute to the recruitment of downstream ESCRT components during autophagy [81,103]. At the cellular level, inhibition of ATG8ylated phagophore closure not only blocks autophagic degradation but can also trigger the activation of signaling molecules recruited to autophagosome formation sites [20–22]. *In vivo*, impaired phagophore closure causes neurological defects and tissue abnormalities that partially overlap with, yet remain distinct from, those observed in core ATG-deficient mice [22,161]. These advances have brought to light several important questions. How does the ESCRT machinery physically constrict and seal the phagophore membrane, and what molecular cues signal that a phagophore has reached the stage at which closure should occur? Besides sensing membrane curvature, how does the N-terminus of VPS37A mediate ESCRT-I targeting during autophagy, and what specific role does the UEVL domain play? How is ESCRT-I recruitment coupled to ESCRT-III filament assembly? Do VPS37A-independent mechanisms for ESCRT recruitment exist in mammals? What factors govern the context-dependent signaling outcomes of ATG8ylated phagophores, and does the VPS37A N-terminus contribute to microautophagy? Finally, what mechanisms underlie the phenotypic differences between VPS37A UEVL mutants and core ATG-deficient mice? Elucidating these questions will advance our understanding of ESCRT-mediated phagophore closure and its physiological significance.

The human VPS37A gene resides on chromosome 8p22, a region frequently affected by large deletions in various solid cancers [172–174]. Most 8p deletions occur as loss of heterozygosity (LOH), suggesting that phagophore closure is unlikely to be completely impaired. Nonetheless, increased numbers of LC3-positive autophagic structures have been detected in immortalized human breast epithelial cells following 8p LOH [173]. Consistently, increased levels of autophagy markers have been observed in tissues from VPS37A UEVL heterozygous mutant mice compared with wild-type mice [22]. Notably, despite the critical roles of autophagy in tumor growth [175,176], down-regulation of VPS37A has been shown to promote tumor progression and correlate with poor patient prognosis [177–184]. Whether this pro-tumor effect is driven by impaired phagophore closure, macroautophagy-independent functions (e.g., endosomal receptor sorting and microautophagy), or a combination of both, remains to be determined. Lastly, VPS37A loss has been shown to not only prime cells for CASP8-mediated cell death but also enhance inflammatory signaling pathways [20,164,165,185]. Future studies are anticipated to test whether activation of these pathways represents a therapeutic vulnerability in cancers harboring 8p loss.

## Open Access

Open access for this article was enabled by the participation of Pennsylvania State University in an all-inclusive *Read & Publish* agreement with Portland Press and the Biochemical Society.

## Abbreviations

BafA1: bafilomycin A1
CRs: cargo receptors
CHMP: charged multivesicular body protein
EPG5: ectopic P-granule autophagy protein 5
ER: endoplasmic reticulum
ESCRT: endosomal sorting complex required for transport
EGFR: epidermal growth factor receptor
ILVs: intraluminal vesicles
LIR: LC3-interacting region
LOH: loss of heterozygosity
MIL: membrane-impermeable ligand
MPL: membrane-permeable ligand
Mod(r): modifier of rudimentary
PE: phosphatidylethanolamine
PI3P: phosphatidylinositol 3-phosphate
SOUBA: solenoid of overlapping ubiquitin-associated domains
UMA: UBAP1–MVB12-associated
UEV: ubiquitin E2 variant
UEVL: ubiquitin E2 variant-like
